# MicroRNA expression profiling of *RAS*-mutant thyroid tumors with follicular architecture: microRNA signatures to discriminate benign from malignant lesions

**DOI:** 10.1007/s40618-023-02023-5

**Published:** 2023-02-07

**Authors:** E. Macerola, A. M. Poma, P. Vignali, A. Proietti, L. Torregrossa, C. Ugolini, A. Basolo, A. Matrone, R. Elisei, F. Santini, F. Basolo

**Affiliations:** 1 Department of Surgical, Medical, Molecular Pathology and Critical Area, University of Pisa, via Savi, 10, 56126 Pisa, Italy; 2 Department of Clinical and Experimental Medicine, University of Pisa, via Savi, 10, 56126 Pisa, Italy

**Keywords:** Thyroid cancer, RAS mutations, microRNA, Follicular-patterned tumors, Molecular markers

## Abstract

**Purpose:**

*RAS* mutations represent common driver alterations in thyroid cancer. They can be found in benign, low-risk and malignant thyroid tumors with follicular architecture, which are often diagnosed as indeterminate nodules on preoperative cytology. Therefore, the detection of *RAS* mutations in preoperative setting has a suboptimal predictive value for malignancy. In this study, we investigated differentially expressed microRNA (miRNA) in benign and malignant thyroid tumors with follicular architecture carrying mutations in *RAS* genes.

**Methods:**

Total RNA was purified from 60 *RAS*-mutant follicular-patterned thyroid tumors, including follicular adenoma (FA), noninvasive follicular thyroid neoplasm with papillary-like nuclear features (NIFTP), papillary and follicular thyroid carcinoma cases (PTC, FTC); 22 *RAS*-negative FAs were used as controls. The expression analysis of 798 miRNAs was performed by digital counting (nCounter nanoString platform).

**Results:**

Comparing *RAS*-mutant and *RAS*-negative FAs, 12 miRNAs showed significant deregulation, which was likely related to the oncogenic effects of *RAS* mutations. Twenty-two miRNAs were differentially expressed in *RAS*-mutant benign versus malignant tumors. Considering the tumor type, 24 miRNAs were deregulated in PTC, 19 in NIFTP, and seven in FTC and compared to FA group; among these, *miR-146b-5p*, *miR-144-3p*, and *miR-451a* showed consistent deregulation in all the comparisons with the highest fold change.

**Conclusions:**

The miRNA expression analysis of follicular-patterned thyroid tumors demonstrated that *RAS* mutations influences miRNA profile in benign tumors. In addition, several miRNAs showed a histotype-specific deregulation and could discriminate between *RAS*-mutant benign and *RAS*-mutant malignant thyroid lesions, thus deserving further investigation as potential diagnostic markers.

**Supplementary Information:**

The online version contains supplementary material available at 10.1007/s40618-023-02023-5.

## Introduction

Thyroid tumors with follicular architecture include benign lesions, low-risk neoplasms and malignant neoplasms with different treatment needs, namely follicular adenoma (FA), noninvasive follicular neoplasm with papillary-like nuclear features (NIFTP), follicular variant papillary thyroid carcinoma (FV-PTC) and follicular thyroid carcinoma (FTC) [1]. These tumors are extremely challenging to identify on fine-needle aspiration (FNA) cytology, and they are often diagnosed as indeterminate nodules [2]. Molecular analysis can be performed on FNA to refine the risk of malignancy of indeterminate nodules. However, both benign and malignant follicular-architecture thyroid neoplasms share the same class of molecular alterations, i.e., *RAS*-like mutations [3]. Alterations in *RAS* genes in thyroid tumors are well-recognized as driver genetic events and include almost exclusively activating point mutations occurring in codons 12, 13, and 61 of *NRAS*, *HRAS*, and *KRAS* genes. These mutations cause a constitutive activation of Ras proteins, leading to a pro-proliferative downstream signaling [4, 5]. The prevalence of *RAS* mutations in follicular-architecture thyroid lesions varies according to the tumor type: the reported frequency ranges from 20 to 30% in FA, and from 40 to 70% in NIFTP; RAS mutations are found in up to 70% FV-PTC and in up to 50% FTC cases [3, 6–8]. Therefore, when detected in indeterminate thyroid nodules, *RAS* mutations indicate the presence of a neoplasm, but cannot unequivocally identify a malignant lesion.

The diagnostic utility of miRNA expression analysis has been extensively explored in thyroid tumors. MiRNAs influence gene expression by inhibiting translation or promoting degradation of mRNA transcripts. MiRNAs can behave both as oncomiR and tumor suppressors, according to their mRNA targets [9]. Over the past 20 years, several miRNAs have been proposed as effective markers for the differential diagnosis of indeterminate thyroid nodules and for the prognostic stratification of tumors; also, miRNAs differentially expressed in a histotype-specific manner have been reported [10]. Across the considerable amount of data on miRNA expression analysis in thyroid tumors, there are few miRNAs widely and consistently reported as deregulated in thyroid cancer, including the overexpression of *miR-146*, *miR-221*, *miR-222*, and *miR-181* [11–13]. To date, only one miRNA-based diagnostic test is available to integrate the cyto-morphological analysis of indeterminate thyroid nodules: the ThyraMIR, a commercial test offered by a private company in the USA (Interpace Diagnostics, Parsippany, NJ, USA). It is performed in combination with ThyGeNEXT test for the screening for oncogenic mutations and fusions. Specifically, only when the ThyGeNEXT is negative or alterations with relatively weak positive predictive value, including *RAS* mutations, are detected, the ThyraMIR test is performed to refine the risk of malignancy of the nodule [14]. The test, which has been recently improved (ThyraMIR v.2), is based on the analysis of expression levels of 11 miRNAs, measured by qRT-PCR. Although in a retrospective study the combination testing showed a high diagnostic accuracy (93%) [15], the ThyraMIR test is not part of the clinical practice of thyroid nodules.

In fact, no miRNA targets have been introduced in the daily management of indeterminate thyroid nodules. In case of *RAS*-positive nodules, the risk of malignancy is highly variable, and there is the need of reliable markers to resolve the uncertainty around these nodules. For this reason, this study aims to investigate miRNAs able to refine the risk of malignancy of *RAS*-mutant thyroid neoplasms.

## Materials and methods

This study has been conducted on retrospectively selected formalin-fixed, paraffin-embedded tissue samples. The study cohort was composed by 60 *RAS*-mutant tumors and 22 *RAS*-negative FAs. *RAS*-mutant thyroid tumors included: 16 FAs, 15 NIFTPs, 19 PTCs (16 follicular variant and three solid variant PTCs), and ten FTCs. The selection of cases was guided by the following criteria: benign and malignant follicular-patterned thyroid lesions positive for *RAS* mutations and available tumor tissue for miRNA analysis. Moreover, 22 *RAS*-negative FAs were retrieved to serve as controls. The characteristics of the sample series are shown in Table 1.

**Table 1:** Clinico-pathological characteristics of 82 thyroid tumors included in the case cohort

	FA *n* = 38	NIFTP *n* = 15	PTC^a^ *n* = 19	FTC *n* = 10	All samples *n* = 82
Age (mean ± SD)	45.4 ± 13.5	44.7 ± 13.1	43.4 ± 14.8	44.1 ± 15.9	45.2 ± 14.2
Sex					
Female	26	13	15	8	62
Male	12	2	4	2	20
Tumor size [cm] (mean ± SD)	3.0 ± 1.2	2.9 ± 1.4	2.8 ± 1.1	2.7 ± 1.2	2.9 ± 1.2
Tumor invasiveness^b^					
Totally encapsulated	38	15	3	\	56
Invasive (PTC) Minimally invasive (FTC)	\	\	12	7	19
Infiltrative (PTC) Widely invasive (FTC)	\	\	4	3	7
Preoperative FNA^c^					
TIR 3A	29	8	13	4	54
TIR 3B	3	3	4	3	13
Not available	6	4	2	3	15
*RAS* status					
Mutant	16	15	19	10	60
Wild-type	22	\	\	\	22

*FA* follicular adenoma; *NIFTP* noninvasive follicular thyroid neoplasm with papillary-like nucelar features; *PTC* papillary thyroid carcinoma; *FTC* follicular thyroid carcinoma; *SD* standard deviation; *FNA* fine-needle aspiration

^a^PTCs included 16 follicular variants and three solid variants

^b^Tumor invasiveness has been reported as follows: FA, NIFTP: absence of invasion by definition. PTC: totally encapsulated, namely follicular variant PTC with complete tumor capsule but not fulfilling NIFTP diagnostic criteria; invasive, PTC shows tumor capsule invasion foci; infiltrative, PTC infiltrates thyroid parenchyma. FTC: minimally invasive and widely invasive, as described in current guidelines [1]; no angioinvasive FTCs were present

^c^According to the Italian consensus for reporting of thyroid cytology [16]

All the available hematoxylin and eosin slides were reviewed to confirm the histological diagnosis following the current guidelines [1]. The presence of *RAS* mutations was assessed in routine practice by real-time PCR with a commercial kit validated for diagnostic use (EasyPGX ready Thyroid, Diatech Pharmacogenetics, Jesi, AN, Italy). The panel includes probes to identify mutations in the following hotspots: *BRAF* p.K601E; *BRAF* p.V600E; *KRAS*, *NRAS*, and *HRAS* codons 12/13 and 61, with no identification of the exact nucleotide change. *TERT* promoter mutation status was also available for 12 out of 19 PTCs, all negative, and for seven out of ten FTCs, four mutated (C228T) and three negative for mutations.

From the same tissue block used for mutation profiling, three 10 µm sections were obtained for miRNA extraction. First, tumor tissue enrichment was performed by manual dissection of areas containing tumor cells; then, total RNA, including miRNA, was purified with a specific kit, following the manufacturer’s instruction (miRNeasy FFPE kit, Qiagen, Hilden, Germany). Total RNA quantity and quality were assessed by the spectrophotometer XPose (Trinean, Gentbrugge, Belgium).

The miRNA expression analysis was performed on 82 samples, 60 *RAS*-mutant and 22 *RAS*-wild-type, by using the nCounter Human v3 miRNA Expression Assay (nanoString Technologies, Seattle, WA, USA) on the nCounter platform (nanoString Technologies), by using 100 ng RNA, as recommended by the manufacturer. Due to an assay upgrade made by the manufacturer, part of cases was analyzed by v3a panel version (*n* = 22), and part by v3b version (*n* = 60); both versions contain probe pairs targeting 798 endogenous miRNAs. Raw data obtained after digital counting from v3a and v3b panels were separately analyzed with the nSolver analysis software (v4.0, nanoString Technologies); data were normalized by using the mean expression of the 100 most expressed miRNAs in the dataset. To select miRNA highly expressed among samples, the mean plus two standard deviations of negative control probes was used as threshold, and targets detected in less than 50% of samples were filtered out. Batch effects due to different version of the panel were removed using the ComBat function of the sva Bioconductor package v.3.20.0. Principal component analysis (PCA) was carried out using the PCAtools Bioconductor package v.2.8.0. Hierarchical clustering was performed using Euclidean distance and Ward clustering method following the procedures of the heatmap3 R package v.1.1.9. Differentially expressed miRNA were computed using the moderated *t* statistics and following the procedures of the limma Bioconductor package v. 3.52.2. Age and sex were used as confounders, and *p* values were adjusted using the Benjamini–Hochberg method. Adjusted *p* values ≤ 0.05 were considered significant. Receiver operating characteristics (ROC) curves analysis was carried out using the pROC R package v.1.18.0. Confidence intervals were estimated by 2000 bootstrap resampling. For data analysis, NIFTP class has been considered as malignant, as it requires surgery.

The set of significantly deregulated miRNAs in benign versus malignant *RAS*-mutant tumors has been entered on DIANA-miRPath v3.0 database (https://dianalab.e-ce.uth.gr/html/mirpathv3, last accessed August 22, 2022), a freely available online tool, which uses both validated and predicted targets to identify pathways controlled by multiple miRNAs, by using Kyoto Encyclopedia of Genes and Genomes (KEGG) annotations [17]. Results were combined by genes union; the options of conservative statistics and correction for multiple comparisons (Benjamini–Hochberg’s FDR) were selected; significance was set as ≤ 0.01.

Statistical analyses (except for pathway enrichment analysis) were performed in R environment (https://www.r-project.org, v.4.1.2, last accessed August 8, 2022).

## Results

### miRNA expression profile of thyroid tumors

After filtering and merging miRNAs from v3a and v3b panels, 128 miRNAs were selected for further analyses and adjusted for batch effect (Suppl. Fig. 1a, b).

**Fig. 1:**
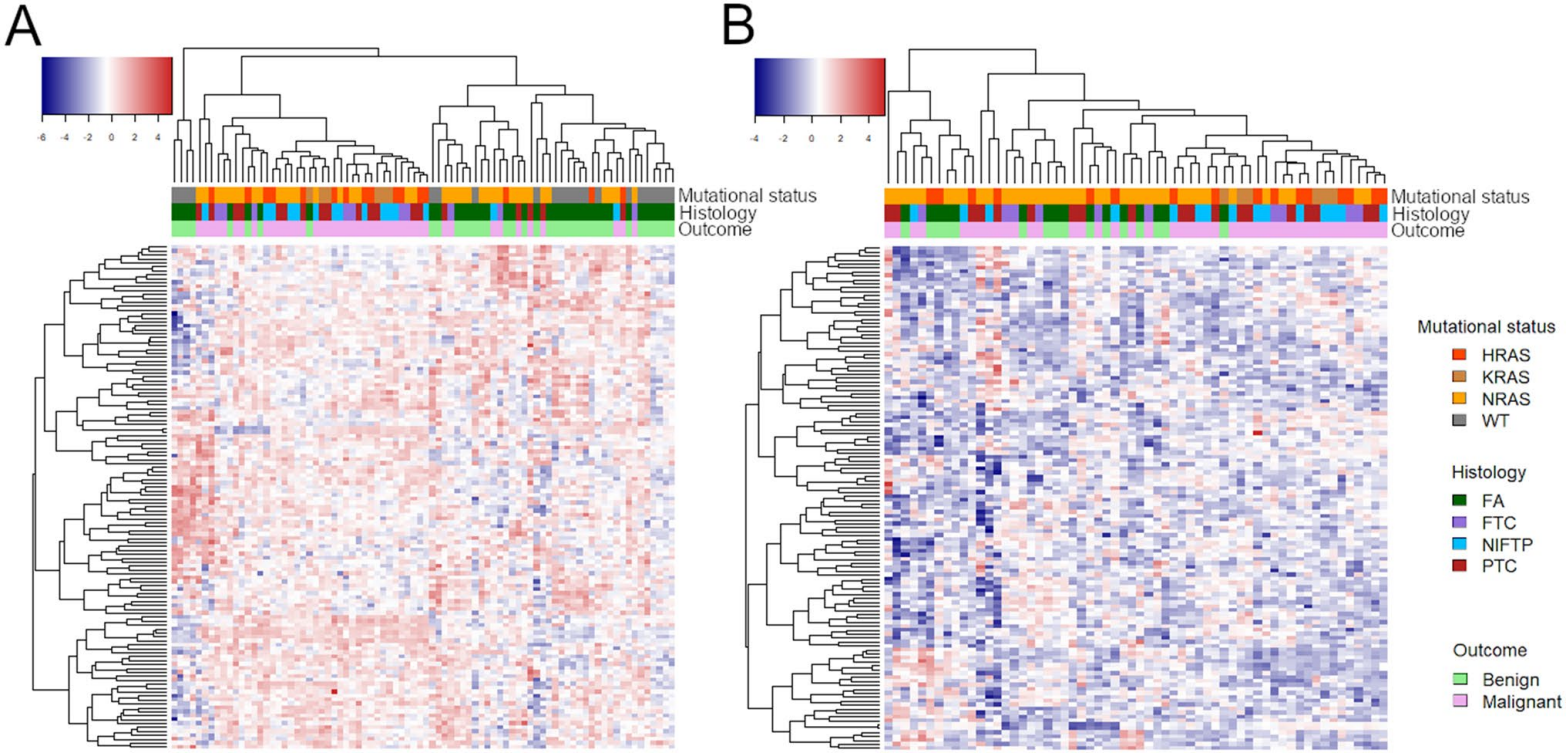
Heatmaps representing the unsupervised clustering of miRNAs and samples. **a** All samples were included (*n* = 82), **b** only *RAS*-mutant samples were included (*n* = 60). Each row represents one miRNA, each column one sample, the colors indicate high (red) and low (blue) gene expression level. Colored sidebars represent mutational status, histological diagnosis, and benign or malignant neoplasm, as it is indicated in the legend

**Fig. 2:**
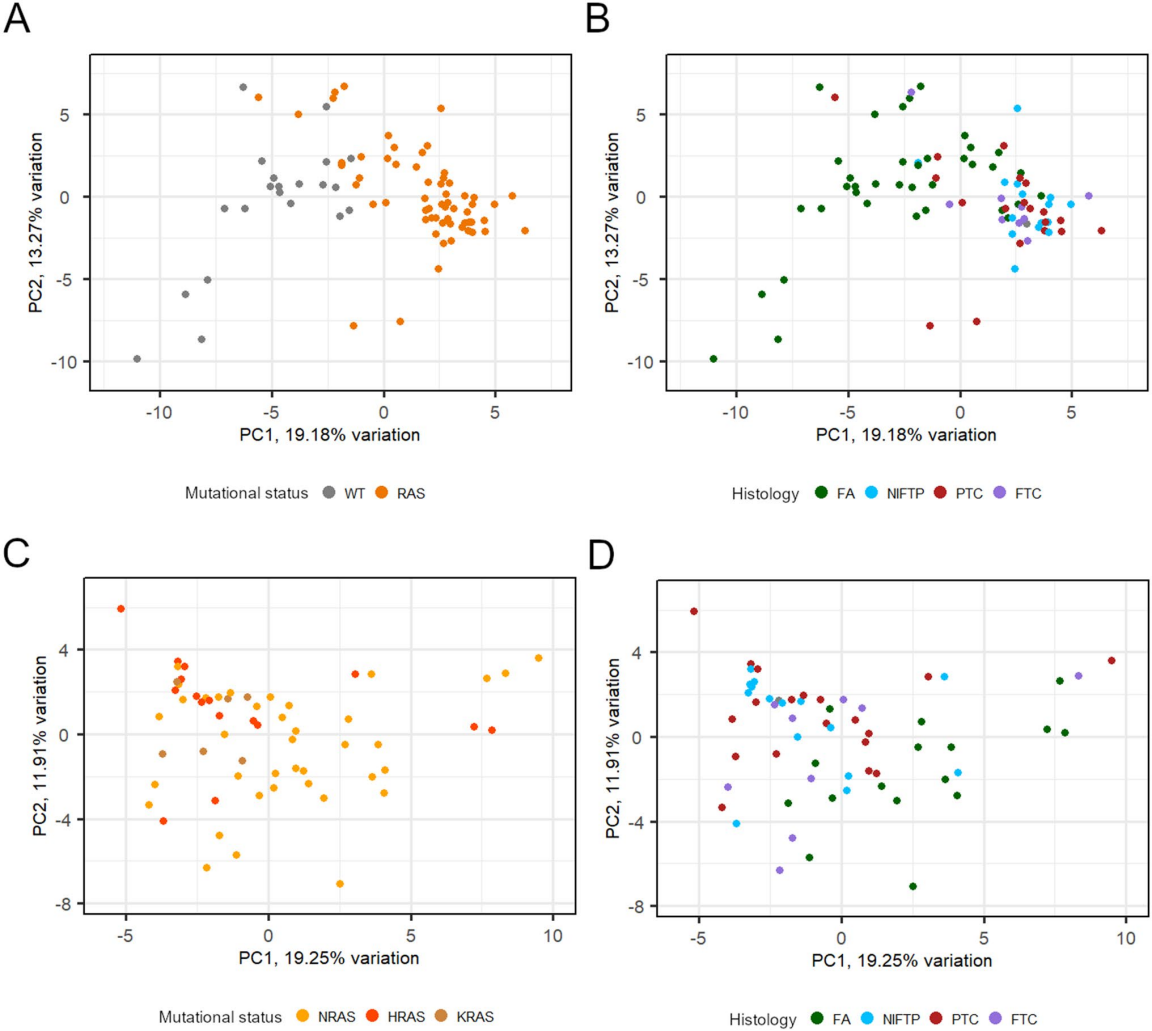
**a**, **b** Principal component analysis of all samples, including *RAS*-negative FAs (*n* = 82), labeled for mutational status and histological class. **c**, **d** Principal component analysis of the 60 *RAS*-mutant tumors, tumors are shown with different colors according to the involved *RAS* gene (*NRAS*, *HRAS*, *KRAS*) and to the histological class

The heatmap shown in Fig. 1a represents the unsupervised clustering of all samples, including the *RAS*-negative FAs, based on the expression of the selected 128 miRNAs. Figure 1b shows the same heatmap after removal of *RAS*-negative samples. The distribution of samples according to *RAS* status and histotype indicates that these factors do have an influence on miRNA expression. This is confirmed by the PCA shown in Fig. 2. In fact, when *RAS*-negative FAs are included, they are grouped together, separately from *RAS*-mutant samples (Fig. 2a). It is interesting to note that *RAS*-mutant FAs are closer to the group of malignant tumors, but still occupy a circumscribed portion of the graph (Fig. 2b). When *RAS*-negative FAs are excluded, samples do not assume a well-defined grouping looking at *RAS* mutation type (Fig. 2c) nor at the tumor type, though FAs occupy the bottom right part of the graph (Fig. 2d).

### miRNAs deregulated in *RAS*-mutant versus *RAS*-negative benign tumors

To identify miRNAs whose deregulation is likely related to *RAS* mutations, a comparison between *RAS*-positive FAs and *RAS*-negative FAs was performed; as shown in Table 2, 12 miRNAs were significantly deregulated.

**Table 2:** Differentially expressed miRNAs between *RAS*-mutant and wild-type FAs

	Deregulated in *RAS*-mutant (*n* = 16) *versus RAS*-negative (*n* = 22) FAs	
miRNA	logFC	Adjusted *P* value
*miR-222-3p*	2.03	< 0.001
*miR-7-5p*	− 2.95	0.004
*miR-221-3p*	1.87	0.004
*miR-221-5p*	1.08	0.004
*miR-19a-3p*	1.10	0.011
*miR-135a-5p*	0.77	0.022
*miR-146b-5p*	1.06	0.041
*let-7a-5p*	− 0.58	0.041
*miR-92a-3p*	− 0.94	0.041
*miR-142-3p*	1.18	0.041
*miR-296-5p*	− 0.72	0.043
*miR-26a-5p*	− 0.49	0.043

The positive or negative sign of base 2 logarithm fold change (logFC) value indicates up-regulation or down-regulation, respectively, in *RAS*-mutant adenoma compared to wild-type adenoma groups

*FA* follicular adenoma; *logFC* base 2 logarithm of the fold change

### miRNAs deregulated in benign versus malignant *RAS*-mutant thyroid tumors

**Fig. 3:**
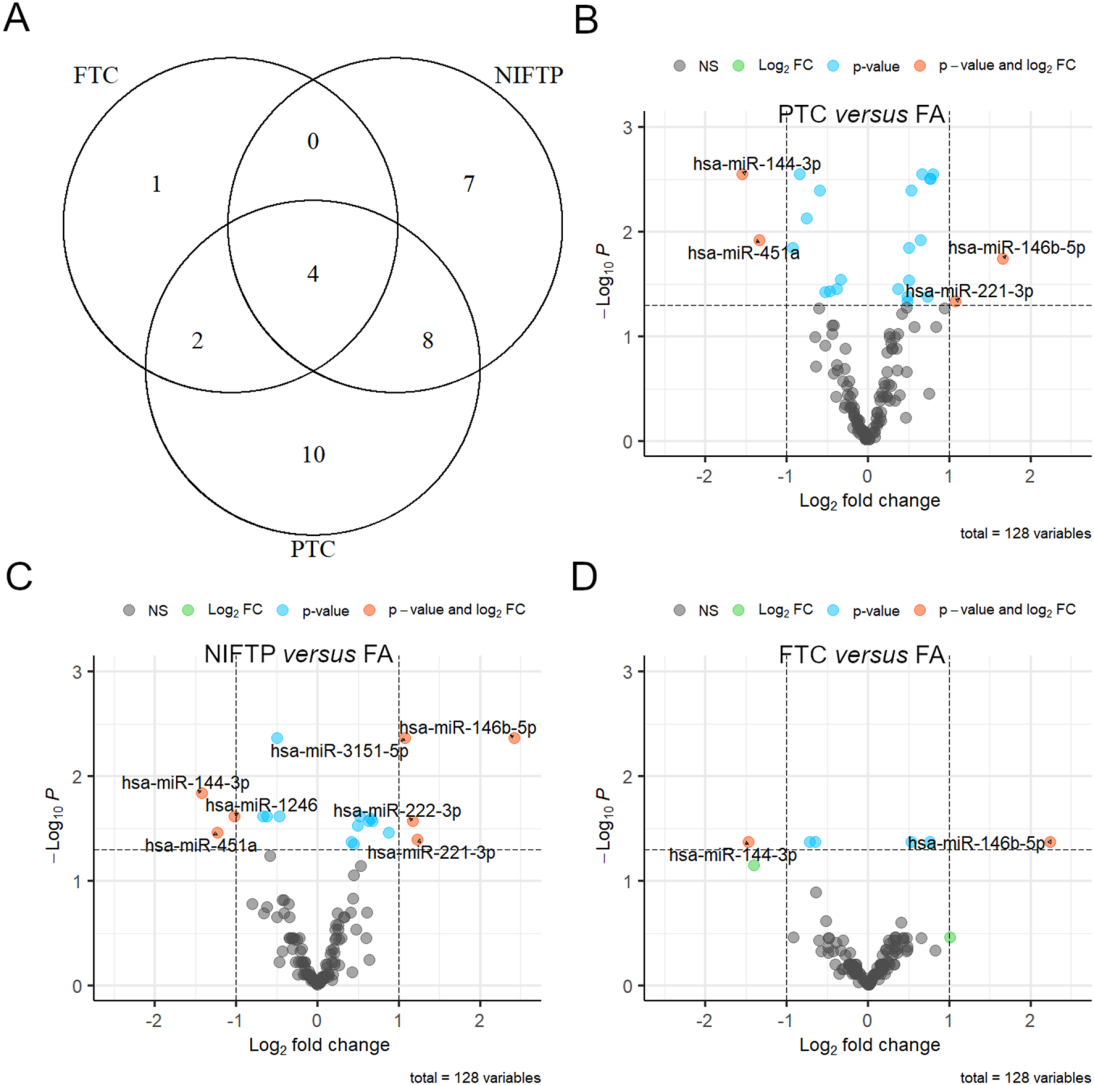
**a** Venn diagram representing the number of significantly deregulated miRNAs in different histological classes. **b**–**d** Volcano plots showing the relative expression (logFC) of miRNAs in PTC, NIFTP, and FTC classes compared to FA group. MiRNAs have been highlighted based on their expression level (green for logFC >|1|), significance level (light blue for *P* value ≤ 0.05), or both (orange for miRNAs showing logFC >|1| and *P* value ≤ 0.05). *FTC* follicular thyroid carcinoma, *NIFTP* noninvasive follicular thyroid neoplasm with papillary-like nuclear features, *PTC* papillary thyroid carcinoma, *FA* follicular adenoma, *NS* not significant, *Log*_*2*_* FC* base 2 logarithm of the fold change

To investigate subtle differences among *RAS*-mutant tumor types, differentially expressed miRNA between benign and malignant cases and among histotypes have been investigated. Comparing all the malignant cases versus *RAS*-mutant FAs, 22 miRNAs were significantly deregulated (12 up- and 10 down-regulated). In addition, 24 miRNAs were deregulated in PTCs, seven in FTCs and 19 in NIFTPs compared to FAs (Table 3). Several miRNAs were deregulated in more than one comparison, as shown in Fig. 3a.

**Table 3:** Significantly deregulated miRNAs in *RAS*-mutant tumors

	Deregulated in malignant versus benign *n* = 22		Deregulated in PTC versus FA *n* = 24		Deregulated in FTC versus FA *n* = 7		Deregulated in NIFTP versus FA *n* = 19	
miRNA	logFC	Adjusted *P* value	logFC	Adjusted *P* value	logFC	Adjusted *P* value	logFC	Adjusted *P* value
***miR-144-3p***	− 1.49	0.001	− 1.55	0.003	− 1.47	0.042	− 1.43	0.014
***miR-146b-5p***	2.03	0.002	1.66	0.018	2.24	0.042	2.42	0.004
*miR-181c-5p*	0.50	0.004	0.54	0.004	0.53	0.042	0.42	0.042
***miR-451a***	− 1.31	0.005	− 1.33	0.012	− 1.40	0.050	− 1.23	0.034
*miR-181b-5p miR-181d-5p*	0.65	0.005	0.76	0.003	\	\	0.63	0.027
*miR-22-3p*	− 0.52	0.005	− 0.59	0.004	− 0.65	0.042	\	\
*miR-99b-5p*	0.65	0.006	0.80	0.003	\	\	0.66	0.025
*miR-324-5p*	0.59	0.009	0.65	0.012	0.76	0.042	\	\
*miR-125a-5p*	0.60	0.009	0.77	0.003	\	\	\	\
*miR-30e-3p*	− 0.35	0.012	− 0.34	0.028	\	\	− 0.50	0.004
*miR-130a-3p*	0.48	0.013	0.77	0.003	\	\	\	\
*miR-497-5p*	− 0.54	0.013	− 0.53	0.037	\	\	− 0.67	0.024
*miR-195-5p*	− 0.51	0.014	\	\	\	\	− 0.62	0.024
*miR-3151-5p*	0.72	0.014	\	\	\	\	1.08	0.004
*miR-30e-5p*	− 0.38	0.014	− 0.38	0.035	\	\	− 0.47	0.024
*miR-221-3p*	1.11	0.014	1.08	0.046	\	\	1.23	0.040
*miR-125b-5p*	0.42	0.024	0.50	0.014	\	\	0.50	0.029
*miR-181a-5p*	0.46	0.030	0.49	0.046	\	\	\	\
*miR-29b-3p*	0.38	0.034	\	\	\	\	0.52	0.024
*miR-143-3p*	− 0.47	0.040	\	\	\	\	\	\
*miR-96-5p*	− 0.51	0.047	− 0.84	0.003	\	\	\	\
*miR-152-3p*	− 0.51	0.047	− 0.75	0.007	\	\	\	\
*miR-1246*	\	\	− 0.92	0.014	\	\	− 1.02	0.024
*miR-1180-3p*	\	\	0.50	0.029	\	\	\	\
*miR-342-3p*	\	\	0.37	0.035	\	\	\	\
*miR-299-3p*	\	\	− 0.47	0.037	\	\	\	\
*miR-92a-3p*	\	\	0.73	0.042	\	\	\	\
*let-7e-5p*	\	\	0.49	0.042	\	\	\	\
*miR-145-5p*	\	\	\	\	− 0.72	0.042	\	\
*miR-135a-5p*	\	\	\	\	\	\	0.68	0.027
*miR-222-3p*	\	\	\	\	\	\	1.17	0.027
*miR-221-5p*	\	\	\	\	\	\	0.87	0.034
*miR-26a-5p*	\	\	\	\	\	\	0.45	0.044

The comparisons are referred to all malignant versus *RAS*-mutant FA cases, and to the single histotypes versus *RAS*-mutant FA group. Only miRNAs with adjusted *P* value ≤ 0.05 have been listed. MiRNAs deregulated in all the comparisons and showing a base 2 logarithm fold change (logFC) value >|1| have been reported in bold

*FA* follicular adenoma; *NIFTP* noninvasive follicular thyroid neoplasm with papillary-like nucelar features; *PTC* papillary thyroid carcinoma; *FTC* follicular thyroid carcinoma; *logFC* base 2 logarithm of the fold change

The 22 deregulated miRNAs in benign versus malignant cases have been used for pathway enrichment analysis on DIANA-miRPath v3.0 tool, and 48 pathways resulted significantly enriched. In Table 4, the top ten pathways showing significant enrichment, ranked by ascending *p* values, are shown.

**Table 4:** List of the top ten significantly enriched pathways (according to Kyoto Encyclopedia of Genes and Genomes, KEGG, database) controlled by the 22 miRNAs deregulated in *RAS*-mutant benign versus malignant tumors

Pathway (KEGG)	adjusted* P* value	Number of target genes	Number of miRNAs
Proteoglycans in cancer	2.2 e–17	133	20
Cell cycle	4.1 e–11	93	20
Renal cell carcinoma	4.4e–10	54	20
Viral carcinogenesis	1.1 e–09	128	20
Pathways in cancer	2.6 e–07	236	20
Prostate cancer	4.0 e–07	67	20
Hippo signaling pathway	4.3 e–07	89	19
Ubiquitin mediated proteolysis	1.0 e–06	93	20
Adherens junction	1.8 e–06	55	20
TGF-beta signaling pathway	4.8 e–06	53	20

**Fig. 4:**
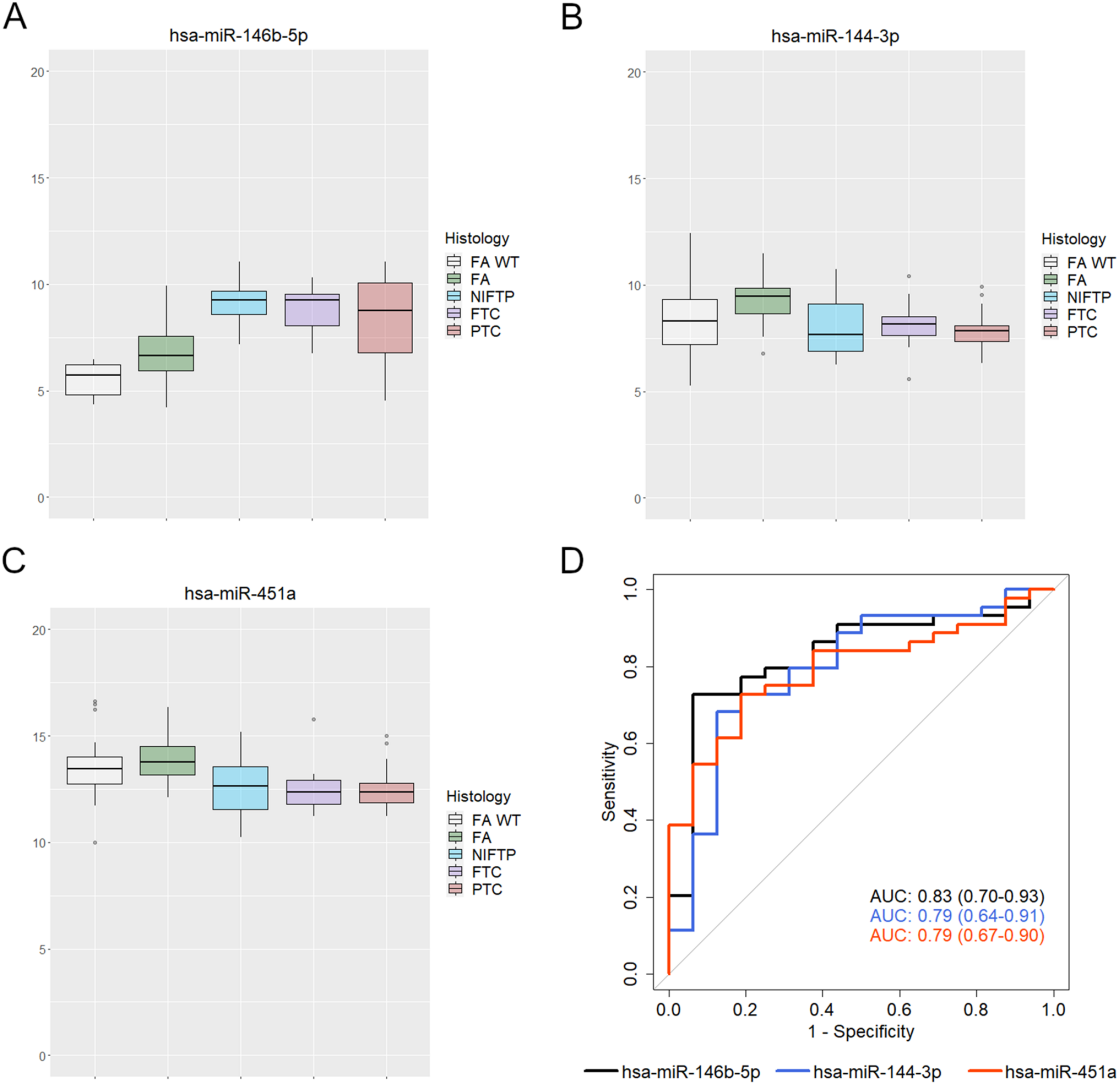
**a–c** Relative expression levels of *miR-146b-5p*, *miR-144-3p*, and *miR-451a* (expressed in log2FC) in all the histological classes. **d** ROC analysis showing the diagnostic performance of the three selected miRNAs. *FA WT* wild-type follicular adenoma, *FA* follicular adenoma, *NIFTP* noninvasive follicular thyroid neoplasm with papillary-like nuclear features, *FTC* follicular thyroid carcinoma, *PTC* papillary thyroid carcinoma

Three miRNAs showed significant deregulation in all the comparisons with an absolute logFC value >|1|; *miR-146b-5p*, *miR-144-3p*, and *miR-451a* (volcano plots in Fig. 3b–d). The expression levels of these miRNAs in all the histological classes are illustrated in Fig. 4a–c; *RAS*-negative FAs have been also included in the graph for a direct comparison. The up-regulation of *miR-146b-5p* appears as a sort of continuum, from *RAS*-negative benign tumors to *RAS*-positive benign and malignant tumors; the expression of *miR-144-3p* and *miR-451a* does not show evident differences between *RAS*-negative and *RAS*-positive benign cases. *MiR-146b-5p*, *miR-144-3p*, and *miR-451a* have been tested by ROC analysis to investigate their potential usefulness in discriminating malignant versus benign *RAS*-positive tumors (Fig. 4d; Table 5). Area under the curve (AUC) was 0.83 (95% CI 0.70–0.93), 0.79 (95% CI 0.64–0.91) and 0.79 (95% CI 0.67–0.90) for *miR-146b-5p*, *miR-144-3p*, and *miR-451a*, respectively.

**Table 5:** Diagnostic performance of *miR-146b-5p*, *miR-144-3p*, and *miR-451a* in discriminating benign versus malignant *RAS*-positive thyroid tumors. All the values are reported with their 95% confidence interval in round brackets

	*miR-146b-5p*	*miR-144-3p*	*miR-451a*
AUC	0.83 (0.70–0.93)	0.79 (0.64–0.91)	0.79 (0.67–0.90)
Specificity	0.94 (0.75–1)	0.87 (0.56–1)	0.87 (0.62–1)
Sensitivity	0.75 (0.61–0.93)	0.75 (0.59–0.98)	0.73 (0.45–0.91)
PPV	0.97 (0.89–1)	0.93 (0.85–1)	0.94 (0.86–1)
NPV	0.58 (0.47–0.80)	0.56 (0.43–0.88)	0.54 (0.39–0.76)

*AUC* area under the curve; *PPV* positive predictive value; *NPV* negative predictive value

## Discussion

Follicular-patterned well-differentiated thyroid tumors represent a morphologically and molecularly homogeneous class of lesions, with a highly heterogeneous clinical course. FA is a benign neoplastic lesion, with clonal development and a well-circumscribed capsule. NIFTP is a low-risk lesion, with cyto-architectural characteristics of FV-PTC but an intact tumor capsule. FV-PTC is often considered a low-risk tumor, at least compared with more aggressive PTC variants, such as tall cell and hobnail variants, and especially when encapsulated [1, 6]. Nevertheless, in a small proportion of cases, FV-PTC can show dedifferentiation, and can cause recurrence and distant metastases [18]. Finally, FTCs show an increasingly aggressive clinical course according to their degree of invasiveness, from minimally invasive to angioinvasive to widely invasive types [6]. All these follicular-patterned tumors, besides the follicular growth pattern, share the same class of molecular alterations, i.e., *RAS*-like mutations. The different level of aggressiveness can be only in part explained by the presence of co-occurring molecular alterations, such as *PIK3CA*, *TP53*, and *TERT* promoter mutations, found in less than 10% of FV-PTCs and in 10–20% of FTCs [7, 19, 20]. In fact, there is controversy about the diagnostic and prognostic role of *RAS* mutations. *NRAS*, *KRAS* and *HRAS* are oncogenes: their activating mutations are known to promote cell growth and proliferation. However, if detected preoperatively on thyroid nodules, *RAS* mutations show a suboptimal positive predictive value for malignancy [21]. Therefore, in this study we investigated miRNA expression profiles in benign and malignant *RAS*-mutant thyroid neoplasms with the aim of identifying miRNAs that could serve as diagnostic markers in presurgical setting, specifically in *RAS*-mutant thyroid nodules, thus helping refine their risk of malignancy.

As a first step, we compared miRNA expression levels in *RAS*-negative and *RAS*-mutant FAs; in this way, we sought to identify miRNAs selectively altered in RAS-positive tumors. In fact, it is known that *RAS* mutations influence miRNA expression profiles in thyroid tumors [22, 23]. However, authors mostly focused on differential miRNA expression between malignant and benign tumors, without considering the presence of *RAS* mutations as a confounding factor [24–26]; on the contrary, several miRNAs are known to be specifically deregulated in *BRAF* mutant thyroid tumors compared to wild-type cases [27, 28]. In previous studies from our group, we demonstrated that wild-type NIFTPs show a benign-like miRNA phenotype, while *RAS*-mutant NIFTPs show a malignant-like miRNA phenotype [29, 30]; in detail, 12 miRNAs were significantly deregulated in mutant versus wild-type NIFTPs (*miR-221-5p*, *miR-221-3p*, *miR-222-3p*, *miR-146b-5p*, *miR-181a-3p*, *miR-28-5p*, *miR-363-3p*, *miR-342-3p*, *miR-1285-5p*, *miR-152-3p*, *miR-25-3p*, *miR-30e-3p*) [30]. Likewise, comparing *RAS*-negative and *RAS*-mutant FAs, herein we report 12 differentially expressed miRNAs, whose deregulation is likely related to *RAS* mutations (*miR-222-3p*, *miR-7-5p*, *miR-221-3p*, *miR-221-5p*, *miR-19a-3p*, *miR-135a-5p*, *miR-146b-5p*, *let-7a-5p*, *miR-92a-3p*, *miR-142-3p*, *miR-296-5p*, *miR-26a-5p*). Interestingly, among these, *miR-221*, *miR-222* and *miR-146b-5p*, well-known to be cancer-related biomarkers [11–13], were significantly up-regulated in mutant FAs. Therefore, activating mutations in *RAS* genes are likely related to the expression of oncogenic miRNAs also in benign thyroid tumors; this could affect the diagnostic performance of molecular classifiers based on miRNA expression profile, resulting in false positive calls. On the other hand, whether these oncogenic miRNAs contribute to promote tumor invasiveness in conjunction with *RAS* mutations is yet to be clarified.

Then, to identify miRNAs with diagnostic value, we analyzed the miRNA expression profile of FAs, NIFTPs, PTCs and FTCs harboring *RAS* mutations. Comparing the FAs versus all the malignant lesions, 22 miRNAs showed significant deregulation. Among these, the most widely described as up-regulated in thyroid cancer are *miR-146b-5b*, *miR-221*, *miR-181*, and *miR99b* [10, 11, 13]; on the other hand, the most well-known downregulated miRNAs are *miR144*, *miR-451a*, *miR-497*, *miR-195*, *miR-143*, and *miR-152* [13]. The analysis of enriched pathways showed that the 22 miRNAs deregulated in malignant *RAS*-positive thyroid tumors play a key role in several cancer-related pathways. It is interesting to note that, in the hypothesis of a diagnostic setting, only one out of 22 differentially expressed miRNAs is currently included in the commercially available miRNA-based diagnostic panel (i.e., *miR-146b-5p*) [15].

The three miRNAs showing significant deregulation in all the comparisons with the highest logFC, i.e., miR-*146b-5p*, *miR-144-3p*, and *miR-451a*, demonstrated good accuracy in distinguishing benign from malignant lesions, with AUC of 0.83, 0.79, and 0.79, and positive predictive value of 0.97, 0.93, and 0.94, respectively. *MiR-146b* is a well-recognized oncomiR in several solid tumors, and it has been often described as a PTC-specific marker. There are even studies exploring therapeutic strategies based on *miR-146b* silencing [31, 32]. Biologically, it is known that *miR-146b* overexpression promotes thyroid cell survival, proliferation, migration, and reduces radioiodine sensitivity through the downregulation of genes such as *ZNRF3*, *SMAD4*, *NIS*, *IRAK1* [30]. In thyroid cancer, the up-regulation of *miR-146b* has been correlated to *BRAF* p.V600E mutation, increased tumor stage and tumor aggressiveness, and higher rate of lymph node metastases [13, 28]. Herein, we demonstrated that up-regulation of *miR-146b-5p* occurs also in benign tumors harboring *RAS* mutations: these findings offer interesting cues on *miR-146b* biological role, but raise also new questions around its diagnostic utility, since it is difficult to establish an absolute cutoff able to reliably predict malignancy. *MiR-144-3p* and *miR-451a*, both downregulated in *RAS* mutant thyroid malignancies in the present study, have been previously described as tumor suppressor miRNAs [23, 33, 34]. The physio-pathological role of *miR-451a* has been investigated in several tumors. In vitro, *miR-451a* inhibits thyroid cancer cells growth, proliferation, epithelial-mesenchymal transition, and induces apoptosis [35, 36]. In thyroid cancer, mainly PTC, *miR-451a* is downregulated, and significant correlation with aggressive clinical-pathological features (tall cell variant, advanced stage, extra-thyroidal extension) has been described [23, 33]. Similarly to *miR-146b*, also *miR-451a* expression seems modulated by the presence of *BRAF* mutations. In fact, significant downregulation of *miR-451a* has been reported in PTCs compared to normal thyroid tissue irrespective of mutation status, but the lower *miR-451a* levels were observed in *BRAF* p.V600E PTCs [23]. In our study, *miR-451a* was not deregulated in wildtype versus *RAS* mutant FAs, but it was significantly downregulated in *RAS*-mutant FAs compared to malignant tumors, thus representing a promising diagnostic marker irrespective of *RAS* mutations. Considering *miR-144-3p*, its overexpression inhibits proliferation, migration, and invasion of thyroid cancer cells in vitro [37]. Although the majority of reports found that *miR-144-3p* is downregulated [10, 13, 23, 34], there are few studies reporting that *miR-144-3p* is overexpressed in thyroid cancer [38, 39]; in one study, the overexpression of *miR-144-3p* was inversely correlated with the transcript quantity of the tumor suppressor gene *PTEN* [38]. In our study, *miR-144-3p* was significantly down-regulated in all the *RAS*-mutant malignant tumors compared to *RAS*-mutant FAs, therefore our report supports the evidence that *miR-144-3p* acts as a tumor suppressor miRNA.

Interestingly, in a study conducted by Knyazeva and colleagues, it was reported that the combination of *miR-146b-5p* and *miR-451a*, which showed reciprocal deregulation, was able to distinguish FA from FTC with an AUC of 0.92 [24]. Using prediction algorithms, the authors hypothesized that the concomitant up-regulation of *miR-146b-5p* and downregulation of *miR-451a* led to the activation of downstream pathways that ultimately promote an invasive behavior in follicular thyroid tumors. Unfortunately, the *RAS* genes status of these tumors was unknown.

As regards miRNA expression profiles among tumor types, the number of deregulated miRNAs was higher in the comparisons between PTCs and FAs (*n* = 24) and between NIFTPs and FAs (*n* = 19), while only seven miRNAs were significantly deregulated in FTCs; some miRNAs showed deregulation in more than one comparison. The fact that NIFTPs and PTCs shared 12 deregulated miRNAs is not surprising, since FV-PTCs and NIFTPs are morphologically similar, with the main difference standing in their degree of invasiveness; on the other hand, the 12 remaining miRNAs deregulated in PTCs and not in NIFTPs might be related to an invasive tumor behavior. In particular, *miR-324-5p* and *miR-22-3p* showed significant up-regulation and down-regulation, respectively, in both PTCs and FTCs versus FAs. Studies conducted in various cancer types, including thyroid cancer, reported that *miR-324-5p* is generally up-regulated [40]. Several authors investigated the biological role of *miR-22-3p*, and described it as a tumor suppressor miRNA [41–43]. In cervical squamous cell carcinoma, *miR-22-3p* downregulation has been associated with metastatic tumor progression [44]; the biological functions of *miR-22-3p* in the context of *RAS*-mutant thyroid tumors has not been investigated.

Of the seven miRNAs deregulated in FTCs compared to FAs, six were in common with PTCs and NIFTPs, and only one was significant in FTCs only, i.e., *miR-145-5p*. The utility of this miRNA in thyroid cancer has been poorly explored. In PTCs, *miR-145-5p* showed downregulation compared to non-tumor tissue, and negative correlation with tumor progression and metastatic spread [45]. However, there is no specific investigation regarding FTCs, thus the biological and clinical role of *miR-145-5p* should be further elucidated.

Our results present some potential limitations. First, it was conducted on a limited set of thyroid cancer types. The cases were retrieved upon careful histological and molecular selection, but it would have been interesting to investigate and compare miRNA expression profiles of aggressive histotypes (poorly differentiated and anaplastic thyroid cancer), and also *RAS*-driven medullary thyroid cancer. Second, only a small set of genetic alterations were screened, and wild-type cases could have harbored uncommon molecular alterations with a potential influence on miRNA expression profiles. Finally, the study was conducted on tumor tissue samples, thus the significance of these results cannot be directly extended to thyroid cytology, and an independent cohort is necessary to validate the diagnostic value of the identified miRNAs.

In conclusion, comparing *RAS*-mutant and *RAS*-negative benign thyroid tumors, we found that *RAS* mutations increase the expression of miRNA with a recognized oncogenic role; therefore, when evaluating miRNA expression profile of thyroid tumors, *RAS* status should be considered as a confounding factor. Moreover, we demonstrated that, besides the morphological similarities and the shared presence of *RAS* mutations, there are miRNAs able to distinguish benign from malignant follicular-patterned neoplasms, with the most promising miRNAs being *miR-146b-5p*, *miR-451a*, and *miR-144-3p*. Moreover, we highlighted that some miRNAs, whose utility in thyroid cancer has been poorly investigated until now, could serve as histotype-specific miRNAs. Our results could help improving the diagnostic performance of already existing molecular classifiers and could support in the selection of miRNA targets for setting up new diagnostic panels.

## Supplementary Information

Below is the link to the electronic supplementary material.

Supplementary file1 (TIFF 142 KB)

## Data Availability

All data generated or analyzed during this study are included in this published article.
