# Supplementary figures and images for: MicroRNA expression profiling of RAS-mutant thyroid tumors with follicular architecture: microRNA signatures to discriminate benign from malignant lesions
Source: J Endocrinol Invest. 2023 Feb 7;46(8):1651–62. doi: 10.1007/s40618-023-02023-5 (PMC10349002; doi:10.1007/s40618-023-02023-5)

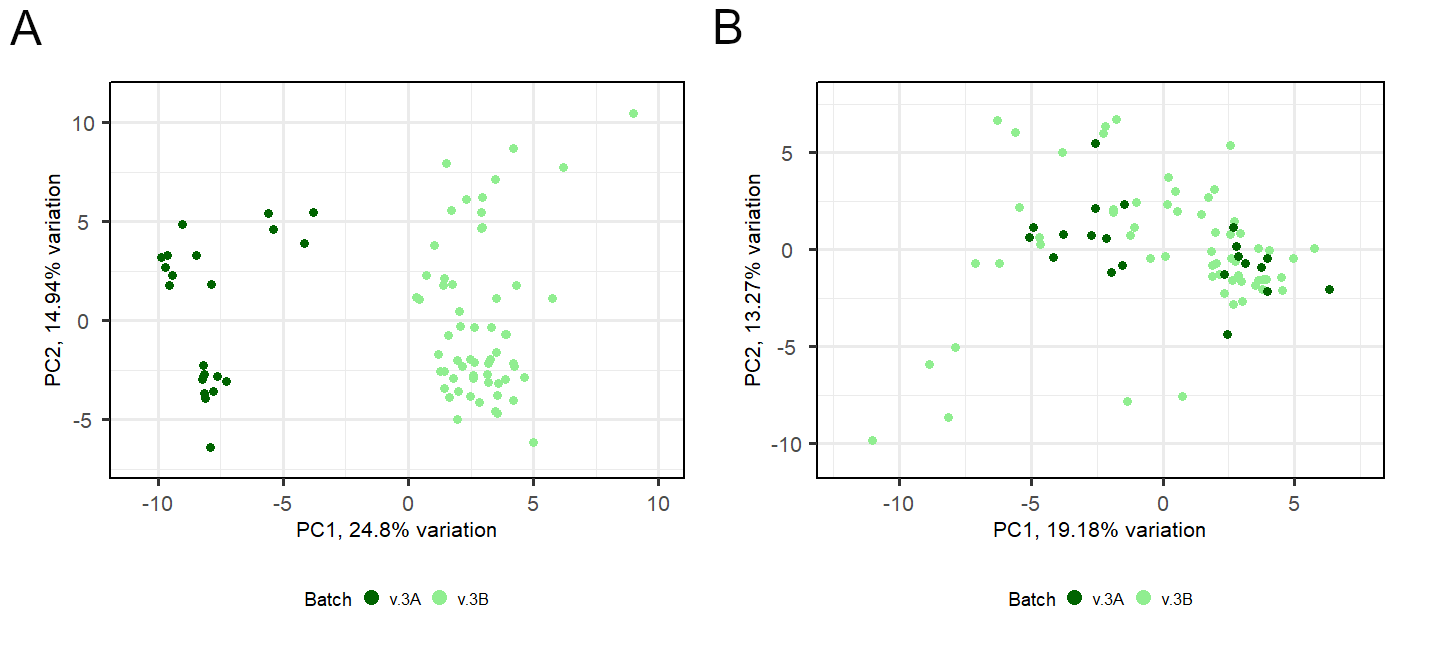

Supplement: Supplementary file 1 — Supplementary file1 (TIFF 142 KB) [file 40618_2023_2023_MOESM1_ESM.tiff]
